# TRPS1 expression in cytokeratin 5 expressing triple negative breast cancers, its value as a marker of breast origin

**DOI:** 10.1007/s00428-023-03535-4

**Published:** 2023-04-03

**Authors:** Szintia Almási, Levente Kuthi, Anita Sejben, András Vörös, Ákos Nagy, Tamás Zombori, Gábor Cserni

**Affiliations:** 1 Department of Pathology, University of Szeged, Albert Szent-Györgyi Medical School, Állomás u. 1, Szeged, 6725 Hungary; 2 Hungarian Centre of Excellence for Molecular Medicine-Semmelweis University, Molecular Oncohematology Research Group, Department of Pathology and Experimental Cancer Research, Semmelweis University, Üllői út 26, Budapest, 1085 Hungary; 3 Department of Pathology, Bács-Kiskun County Teaching Hospital, Nyíri út 38, Kecskemét, 6000 Hungary

**Keywords:** Triple-negative breast cancer, TRPS1, CK5, Immunohistochemistry

## Abstract

The lack of oestrogen receptor, progesterone receptor and human epidermal growth factor receptor-2 expression in breast cancer (BC) is the basis for the categorization of the tumour as triple negative breast carcinoma (TNBC). The majority of TNBCs are aggressive tumours with common metastases and decreased expression of markers that could help in identifying the metastatic lesion as of mammary origin. Breast markers, such as gross cystic disease fluid protein-15 (GCDPF-15), GATA binding protein 3 (GATA3), mammaglobin (MGB) and SOX10, are not uniquely specific to BC. Our aim was to evaluate trichorhinophalangeal syndrome type 1 (TRPS1) protein as a breast marker in a series of cytokeratin-5-expressing TNBC, mostly corresponding to basal-like TNBCs, previously characterized for the expression of other breast markers. One hundred seventeen TNBCs in tissue microarrays were immunostained for TRPS1. The cut-off for positivity was ≥ 10%. The reproducibility of this classification was also assessed. TRPS1 positivity was detected in 92/117 (79%) cases, and this exceeded the expression of previously tested markers like SOX10 82 (70%), GATA3 11 (9%), MGB 10 (9%) and GCDFP-15 7 (6%). Of the 25 TRPS1-negative cases, 11 were positive with SOX10, whereas 5 to 6 dual negatives displayed positivity for the other makers. The evaluation showed substantial agreement. Of the five markers compared, TRPS1 seems the most sensitive marker for the mammary origin of CK5-expressing TNBCs. Cases that are negative are most often labelled with SOX10, and the remainder may still demonstrate positivity for any of the 3 other markers. TRPS1 has a place in breast marker panels.

**Supplementary Information:**

The online version contains supplementary material available at 10.1007/s00428-023-03535-4.

For therapeutic and prognostic purposes, breast cancer (BC) is often classified on the basis of oestrogen receptor (ER), progesterone receptor (PR) and human epidermal growth factor receptor-2 (HER2) expression. The lack of immunohistochemical evidence of these proteins in tumour cells leads to the categorization of the tumour as triple negative breast carcinoma (TNBC). TNBCs are often high-grade tumours (not uncommonly even devoid of obvious glandular differentiation) with poor prognosis that overlap with carcinomas classified as basal-like on the basis of gene expression profiling [1–3], especially if they express cytokeratin 5 (CK5) and/or epidermal growth factor receptor [4]. However, the two categories are not identical, as TNBCs can be subdivided according to gene expression into distinct categories [5–7], and also include rare tumours with a relatively indolent behaviour [8]. Even with this in mind, the majority of TNBCs are aggressive tumours with common metastases and decreased expression of markers that could help identifying the metastatic lesion as of mammary origin. Per definition, TNBCs lack ER, PR and HER2 expression that are not specific markers of BCs, but as the majority of BCs express them in some combination, they might be helpful in orienting the attention to the breast primary in cases of distant metastases. Common breast markers include GCDPF-15 (gross cystic disease fluid protein 15) GATA3 (GATA binding protein 3), mammaglobin (MGB) and SOX10 (SRY-box transcription factor 10), but neither of these are uniquely specific to BC.

GCDFP-15 is also a marker of apocrine differentiation if diffusely expressed, and therefore obviously labels a subset of TNBC (and skin appendage tumours), that show apocrine differentiation [7, 9, 10]. On the other hand, non-apocrine breast cancers may also be stained with this antibody, and this phenomenon may serve as evidence of breast origin in metastatic cases. Besides cutaneous tumours, other neoplasms that have been reported to be GCDFP-15 positive include salivary gland adenocarcinomas [11, 12] and prostatic adenocarcinoma [12, 13].

As breast marker, MGB has proven to be more sensitive than GCDFP-15, but is also expressed in other tumours, mainly in endometrial carcinomas, but rarely also in some sweat and salivary gland tumours, pancreatic and ovarian carcinomas and other tumours including some melanomas [9].

GATA3 is probably one of the most sensitive markers of BC [14], but is expressed in numerous other types of tumours, including the majority of urothelial carcinomas, and other cancers [15]. As GATA3 is a key component of the ER-alpha-GATA3-FOXA1 transcriptional network [16], it is logical that TNBCs might have this marker expressed less frequently than luminal or luminal-like BCs. Indeed, our previous analysis of CK5-expressing TNBCs suggested that GATA3 was expressed in 71% of the 115 cases tested, but only 23 cases (20%) expressed this protein in > 5% of the tumour cells [17]. This relatively low proportion of tumour cells and the often weak staining make GATA3 less reliable in the context of CK5-expressing TNBCs, even though it seemed to be the best marker among GATA3, MGB, GCDFP-15 and NYBR1, the latter of which only labelled 6% of the CK5+ TNBC cases with only 3% showing > 5% staining [17], in keeping with a previous report correlating its expression to ER positivity and inversely correlating it to EGFR [18].

In contrast to the previous markers, SOX10 turned out to be a reliable marker of TNBCs which also labels myoepithelial cells, and shares high specificity for melanocytic tumours [19–21]. Other SOX10 positive tumours include salivary and skin adnexal gland tumours [22, 23]. In keeping with the above, SOX10 outperformed GATA3, MGB and GCDFP-15 as a mammary origin marker in TNBC [24, 25].

TRPS1 (trichorhinophalangeal syndrome type 1) is a transcription factor of the GATA family, and has been found to be a relatively specific marker of breast cancers through TCGA (The Cancer Genome Atlas) data mining [26]. It is not only expressed in a high proportion of ER-positive or HER2-positive BCs, but also in TNBCs of different types, including metaplastic carcinomas, whereas its expression in other cancer types was absent or negligible [26, 27]. In the present study, we examined TRPS1 expression in a subset of TNBC most likely to overlap with a basal-like phenotype on the basis of CK5 expression, and compared this with the expression of other breast markers.

## Material and methods

Immunostaining was done on tissue microarrays constructed from TNBC with CK5 expression as described earlier [17]. Antibody details for breast marker other than TRPS1 were reported previously and are summarized in Table 1. Staining results for GATA3, MGB, GCDP-15 and SOX10 were used from previous work [17, 25], but the cut-offs for positivity were also tested as ≥ 10% rather than > 5% (as used in several previous works) as this higher percentage allows easier detection and interpretation.

**Table 1:** Antibodies used for marker assessment

Antibody	Clone	Source	Dilution
TRPS1	Polyclonal rabbit	Invitrogen, Waltham, MA	1:250
SOX10	A-2	Santa Cruz, Dallas, TX	1:500
GATA3	HG3-31	Santa Cruz, Dallas, TX	1:50
MGB	1A5	Biocare, Concord, CA	RTU
GCDFP-15	23A3	Cell Marque, Rocklin, CA	1:200

*RTU* ready to use

For TRPS1, nuclear staining of any intensity in at least 10% of the tumour cells was interpreted as positive. Six pathologists (SzA, LK, AS, AV, TZ, GC) evaluated the immunohistochemistry with TRPS1. All participants have classified the cases as positive or negative using this cut-off value, to estimate the reproducibility of interpretation. Majority opinions were selected for labelling a case negative or positive.

In addition, we studied 6 breast cancer cases demonstrating HER2 positivity and CK5 expression (basal HER2 carcinomas by immunohistochemistry (IHC)). Among these, half were classified as basal-like and half as HER2-enriched on the basis of the expression of the Prediction Analysis of Microarray 50 (PAM50) gene expression pattern. This was determined from formalin-fixed paraffin-embedded tissues by the Breast Cancer 360™ Panel (Nanostring, USA) on the NanoString nCounter® FLEX platform (Nanostring, USA) following the manufacturers’ instructions.

Reproducibility was assessed by ONEST (observers needed to evaluate subjective tests) [28–31] using an open-source software [31, 32] and kappa statistics [33]. Ninety-five percent confidence intervals (CIs) of proportions were calculated with the VassarStats software (vassarstats.net).

This study was approved by the Human Investigation Review Board, University of Szeged.

## Results

**Fig. 1:**
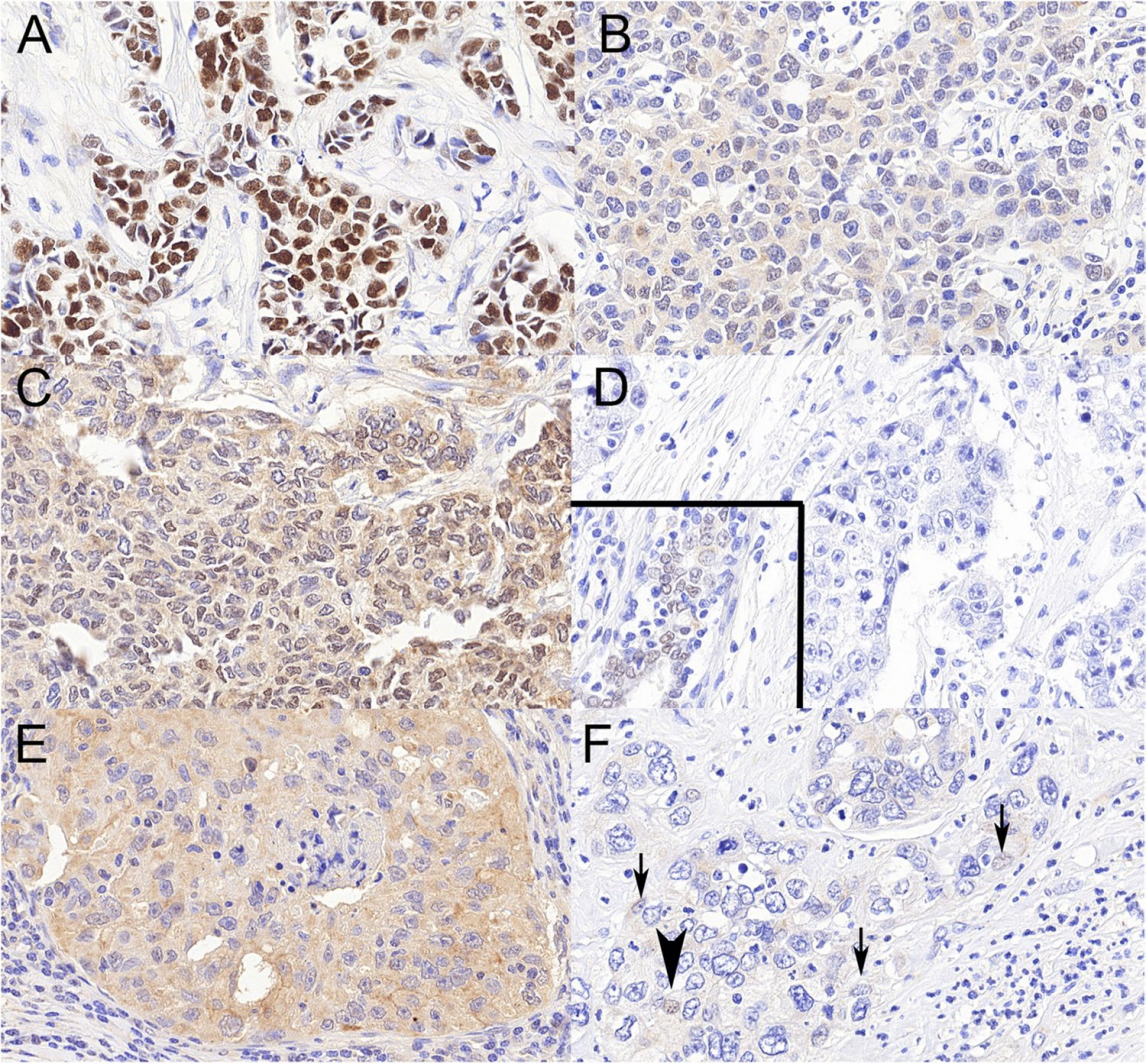
Examples of TRPS1 positive (ABC) and negative (DEF) immunostainings. **A** Obvious positive staining with strong nuclear labelling and no cytoplasmic background. **B, C** Weak nuclear staining in > 10% of the cells interpreted as positive or negative by 50% of the observers; note the relatively strong background cytoplasmic staining. **D** Completely negative reaction, inset showing positive staining in a normal duct of the same TMA core. **E** A case with strong cytoplasmic background staining ignored due to the lack of nuclear staining. **F** The case with about 5% of the nuclei staining weakly (bold arrowhead) or in a barely visible fashion (arrows). All ×40, TRPS1

Of the 120 cases, only 117 had evaluable samples. The majority (*n* = 112) of the tumours were invasive breast carcinomas of no special type, inclusive of 6 cases with medullary pattern and 2 with mixed invasive micropapillary component, but a few metaplastic carcinomas were also part of the tumours investigated, 4 with squamous and 1 with heterologous mesenchymal differentiation. Examples of TRPS1 immunostains are shown in Fig. 1.

For the ONEST plot analysis, all permutations (6! = 720) of the observers were used, rather than only 100 randomly chosen as suggested by the first descriptions and uses [28, 29]. The main descriptors of ONEST included 72.6% overall percent agreement, 19.7% bandwidth (greatest difference in rating by 2 observers) and a minimum of 4 observers needed to assess reproducibility. The Cohen’s kappa coefficient was 0.67, reflecting substantial agreement [33]. Majority opinions were used for categorization as positive or negative, and for the two cases with 50–50% split of opinions, revision of the slides was done to categorize the case as positive (these are illustrated in Fig. 1B, C)

**Fig. 2:**
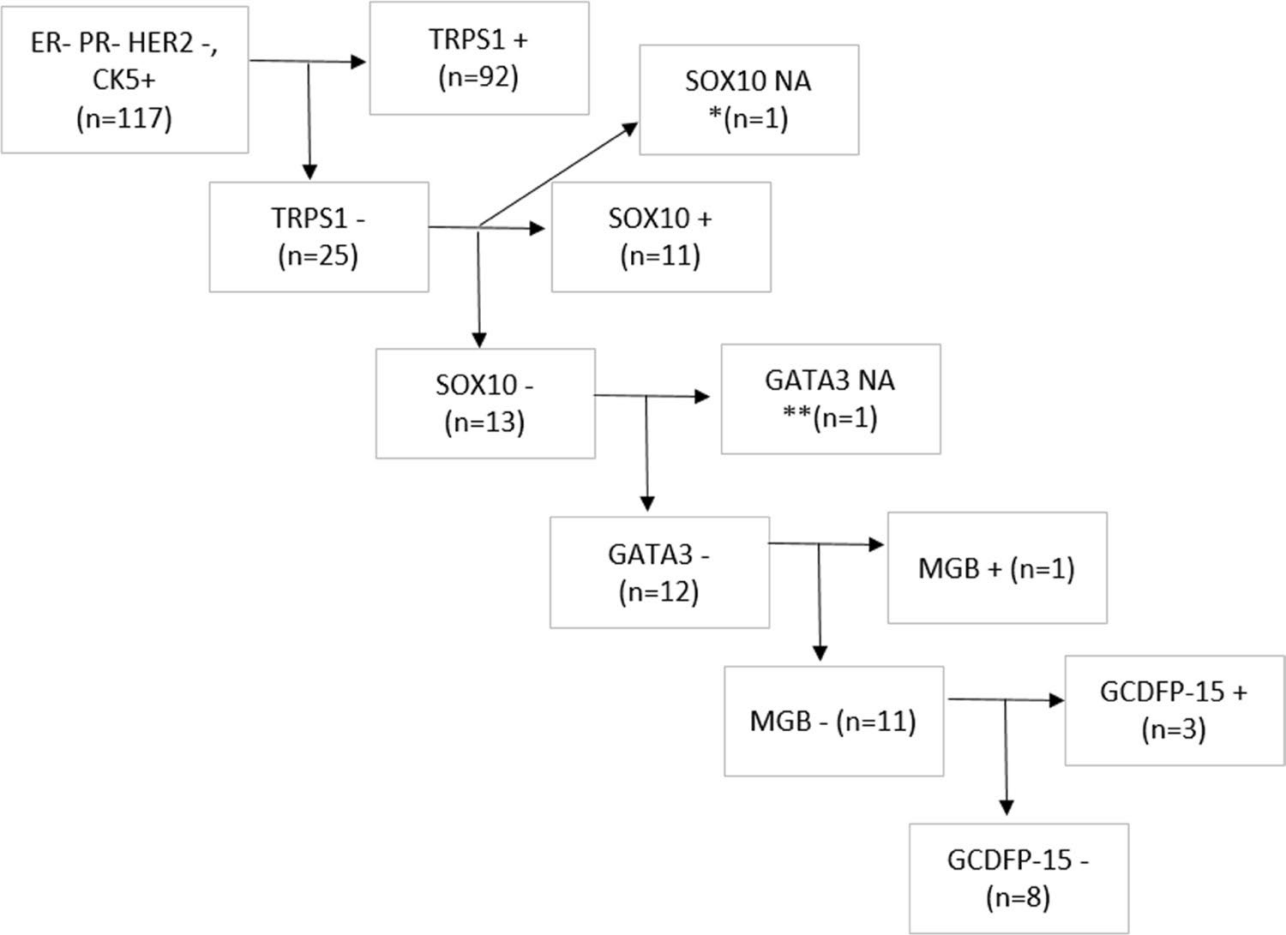
Hierarchical labelling of CK5+ TNBC cases with TRPS1, SOX10, GATA3, MGB and GCDFP-15 (*none of the other stains were evaluable; ** the case had no GATA3 and MGB slide available, but 80% of tumour cells were GCDFP-15 positive); NA, not available The order of the markers follows their positivity rate from highest to lowest from left to right

Of the 117 evaluable tumour samples, 92 samples (79%; 95% CI 70–85%) showed nuclear staining with TRPS1 IHC in 10 to 100% of tumour cells. Generally, a diffuse staining was seen; 78/92 cases (85%; 95% CI 75–91%) showed ≥ 50% nuclear labelling, and this was the case in 3/5 metaplastic carcinomas, of which the remaining two (with squamous metaplasia) turned out to be negative. The remainder, i.e., 25 samples, were completely negative, or showed at times strong cytoplasmic staining without nuclear labelling with the exception of 1 case which showed very weak labelling in about 5% of nuclei (Fig. 1D–F). Discrepant interpretations were generally seen in cases of non-diffuse labelling. As concerns the other markers, their number (rate) of positivity (with the same 10% cut-off) were as follows: SOX10, 82 (70%; 95% CI 61–78%); GATA3, 11 (9%; 95% CI 5–17%), MGB 10 (9%; 95% CI, 4–16%) and GCDFP-15, 7 (6%; 95% CI 3–12%). This order was taken into account when organizing the IHC markers in hierarchy (Fig. 2).

Of the 25 TRPS1-negative cases, 11 samples were positive, and 13 were negative with SOX10. To continue this line, out of 13 TRPS1 and SOX10 dual-negative cases, none showed GATA3 positivity, and one was not assessable. Of the 12 TRPS1, SOX10 and GATA3 triple-negative cases, only 1 was positive with MGB. The remaining 11 cases were divided into 3 GCDFP-15-positive and 8 GCDFP-15-negative cases (Fig. 2, Supplementary figure 1).

**Fig. 3:**
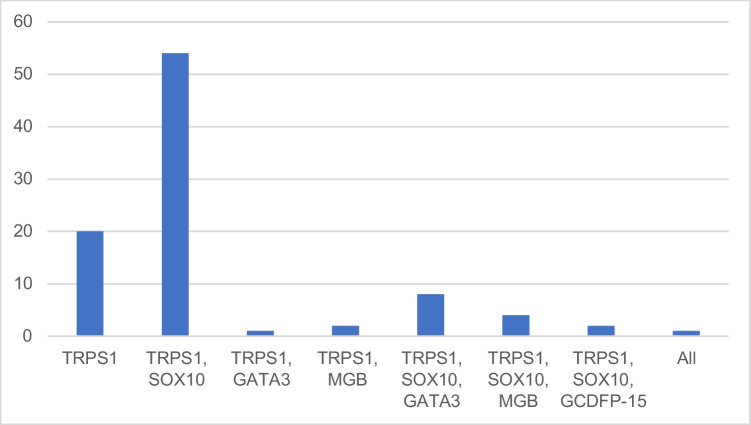
Distribution of positive stainings with different breast markers in the 92 TRPS1-positive cases (number of cases on axis *y*)

Of the 92 TRPS1-positive cases 20 were positive with only this marker (22%, 95% CI 14–32%); the rest showed dual or triple positivities, and a single case was positive for all 5 markers (Fig. 3).

**Fig. 4:**
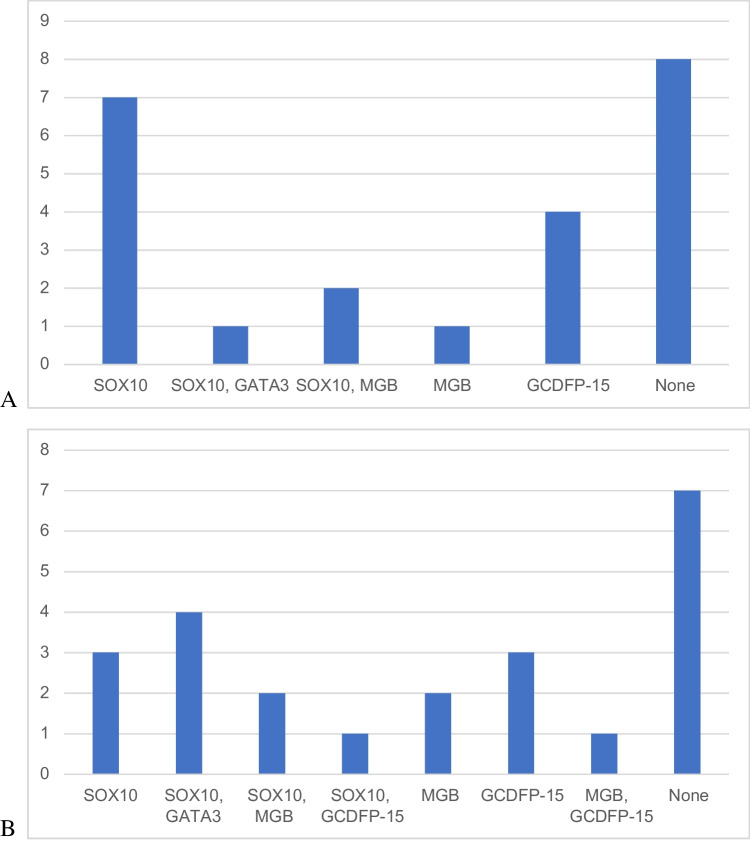
Distribution of positive stainings with different breast markers in the 23 TRPS1-negative cases with available data for the rest of the markers. **A** All markers with 10% cut-off. **B** Using 5% positivity cut-off for GATA3, MGB and GCDFP-15. (Number of cases on axis *y*)

The 25 TRPS1-negative cases displayed various staining with other breast cancer markers. One case had no available results for the rest of the markers, and one had only positive SOX10 results available. With a general 10% cut-off for positivity, 7 cases were positive with SOX10 only. One case showed dual positivity with SOX10 and GATA3. Two cases were positive with both SOX10 and MGB. One case was positive with MGB only. Neither of the cases was positive with just GATA3. Four cases were positive with GCDFP-15. Eight cases were negative with all of the examined breast cancer markers (Fig. 4A). As the proportion of positive TNBC cases is much influenced by the cut-off values of GATA3, MGB and GCDFP-15, we have also assessed a 5% cut-off (only for these 3 markers) used by several other studies to see the labelling of our TRPS1 negative cases. The results are displayed in Fig. 4B.

The 6 cases showing HER2 positivity along with CK5 positivity (independently of being identified as HER2-enriched or basal-like) were all positive for TRPS1 and, in keeping with SOX10 being a marker of TNBCs, were negative for SOX10. The other markers were not tested.

## Discussion

GCDFP-15, MGB and GATA3, as the first reported breast markers, all show less sensitivity in TNBCs [34]. Sensitivities vary according to the proportion of staining cells and intensities used as cut-off values for distinguishing negative and positive cases. With low cut-offs, such as any staining, more tumours turn out to be positive (e.g. primary breast carcinomas of mixed phenotype being positive in 94% (GATA3), 83% (GCDFP-15) and 89% (MGB) [14]), but these become less frequent with higher cut-offs. Indeed, our previous analysis of CK5-expressing (IHC defined basal-like) carcinomas demonstrated 82%, 30% and 23% positivity with any staining for GATA3, MGB and GCDFP-15, respectively, but this decreased to 23%, 12% and 9% by applying a cut-off as low as > 5% staining [17]. When applying a more readily perceived cut-off of at least 10%, the proportions went down to 9%, 9% and 6%, respectively. Low percentage of weakly staining cells always cast some doubt about the interpretation of the given IHC slides, despite the fact that even low positivity rates may point to breast cancer origin in relation to TNBCs. Diminishing the cut-off to 5% did not greatly impact on SOX10 and TRPS1 positivity rates, 82 vs 86/117 and 92 vs 93/117, respectively.

The results may also be different by antibody clones. For example, GATA3 clone HG3-31 was shown to be less sensitive to label TNBCs than L50-823 [35]; therefore, the use of other clones of antibodies may alter the results.

Of the breast markers, SOX10 has been shown to have the highest sensitivity and specificity (compared to GATA3, MGB and GCDFP-15) to discriminate between TNBC and lung adenocarcinomas to reflect the differential diagnosis of TNBC metastatic to the lung [24].

Even with the potential variation with different clones of antibody in mind, literature data and the presented results indicate that SOX10 and TRPS1 are more sensitive breast markers than GATA3, MGB or GCDFP-15 for TNBCs, including their subset expressing CK5 and overlapping in phenotype with squamous carcinomas. In this series, TRPS1 was the most expressed breast marker, followed by SOX10, GATA3, MGB and GCDFP-15; the sensitivity of TRPS1 was 0.86 whereas that of SOX10 was 0.69 (with a 10% cut-off, and these values would have been 0.87, and 0.72, respectively with a 5% cut-off). We have not investigated other tumour types; therefore, the specificity of TRPS1 could not be determined directly, but on the basis of the limited data available, this is also a rather specific marker.

In 2020, Ai et al. examined 31 different solid tumour types through TCGA data mining, and found TRPS1 a protein specific for breast carcinoma. The 479 cases of various types of breast cancers they analysed with IHC showed a high proportion of staining in ER/PR positive (95% of 176), HER2 positive (79% of 67) and TNBCs (81% of both 52 metaplastic and 184 non-metaplastic cases), being more sensitive than GATA3 for this latter subset. An evaluation of altogether 1234 different solid tumours from different organs revealed TRPS1 to be specific, too. Of carcinomas of the bladder, lung, ovary, salivary duct, pancreas, colon, stomach, kidney and thyroid as well as melanomas, strong expression was only seen in 2/77 pulmonary squamous cell carcinomas, 2/165 serous and 1/86 non-serous ovarian adenocarcinomas and 7/143 salivary duct carcinomas. Lesser intensity staining was also identified in a significant minority of the same tumour types (up to 22% in squamous carcinoma of the lung and 19% in salivary duct carcinomas), and very rare cases of pulmonary adenocarcinomas (3/122), 1/144 pancreatic adenocarcinomas and 1/40 melanomas also showed low to intermediate labelling. They concluded as TRPS1 is a highly sensitive and specific marker for breast carcinomas, including TNBC [26].

Similarly, Parkinson et al. have also found TRPS1 to diffusely (≥ 50% of the cells) stain the majority of HER2-positive cancers (91% of 64 cases) and TNBCs (87% of 76 cases), with a minority of tumours being labelled to a lesser degree, and only 3 and 6 cases showing < 10% labelling, i.e. being below the cut-off used in the present study. In addition, in other types of cancer investigated including colorectal (*n* = 208), hepatocellular (*n* = 208), endometrial (*n* = 93) carcinomas, cholangiocarcinomas (*n* = 106) and pulmonary adenocarcinomas (*n* = 49), only 3 and 1 of the latter two showed > 1% staining, which again suggests that TRPS1 is not only sensitive, but also specific for breast carcinomas in general, and TNBC is no exception to high sensitivity [27].

Yoon et al, have investigated primary or metastatic TNBCs of no special type or lobular type, and only 1/151 turned out to be negative with TRPS1, the rest demonstrated > 10% staining, with the majority showing > 50% [35]. They also analysed the staining in 141 metaplastic carcinomas, of which only 7 were negative, and 11 showed < 10% staining, i.e., the majority of metaplastic TNBCs were also TRPS1 positive [36], which points to a minor overlap with squamous carcinoma labelling described by Ai et al. [26]. The proportion of staining cells of their cases is in keeping with our results suggesting that most cases stain in at least 10% of the cells.

Du et al. have also analysed several subsets of breast carcinomas with TRPS1, and found that altogether, only 8% of 1201 breast carcinomas were completely negative for this marker, and further 13% were weakly positive (≤ 10% staining, i.e., negative according to our criteria) [37]. In contrast to the previously cited studies, metaplastic carcinomas were those that showed the highest positivity rate (129/140, 92% demonstrating over 10% staining), and non-metaplastic TNBCs being the less frequently positive (in 69% of 144 cases).

There was substantial agreement between the observers in rating the cases as positive or negative with TRPS1, and the ONEST analysis suggested over 70% overall agreement. The number of observers needed to reliably reflect reproducibility was 4, and this is more than for ER or PR, but less than for Ki67 with a similar 10% cut-off, all being nuclear staining proportions evaluated [29]. Although cases as the one shown in Fig. 1F with low percentage of the cells staining only weakly may escape detection and were considered negative with our 10% cut-off value, it must be remembered that weak and low proportion of cells staining does not exclude mammary origin, and is less common with TRPS1 than with other markers. Less than perfect agreement was also related to cases with weaker nuclear and/or cytoplasmic staining as shown in Fig. 1B, C.

It is well known that IHC is only a surrogate approach to reflect gene expression-based intrinsic subtypes of breast cancer [38]. Therefore, CK5 expression only increases the likelihood of a TNBC to be of the basal-like subtype [4], and cannot substitute just approximate the results of gene expression profiling. This is why we were also interested in the TNBC breast marker expression of 6 cases with CK5 expression that did not satisfy the category of TNBC, but expressed HER2 on IHC, as 3 of these were classified as basal-like on the basis of mRNA expression. The numbers are obviously low, but all were positive for TRPS1, in keeping with this marker being a useful pan-breast cancer marker, including TNBCs and basal-like cancers.

Based on our results, of the five markers compared, TRPS1 seems the most sensitive marker for the mammary origin of CK5-expressing TNBCs (most likely to coincide with basal-like breast carcinomas), and might be best exploited in the metastatic setting. Cases that are negative are most often labelled with SOX10, whereas the dual-negative subset may still be positive for one of the additional markers. In our series, interestingly, GATA3 had no additive value (neither with 10% nor with 5% cut-offs for positivity), and GCDFP-15 was the most expressed marker in this minority of TRPS1 and SOX10 negative cases. The reproducibility of the evaluation showed substantial agreement in our current study, which means it is an easily assessable marker.

The clarification of whether a triple-negative carcinoma in the breast is a primary breast carcinoma or not is dependent on several contextual features, like the presence of corresponding in situ carcinoma, the morphology matching histological types of breast cancers (including rare salivary gland-like tumours) versus unconventional morphologies, and these may obviate the need for breast marker testing. Core biopsies may be less representative, and may require more frequent testing. Of course, both in the primary and the metastatic setting, relevant clinical history is of prime importance. But when breast marker testing becomes a need, a panel of markers is best to be used, as even the least sensitive marker may be the only positive one. On the basis of our results and the cited literature data, we suggest that TRPS1 has a good place in these panels.

## Supplementary information

ESM 1: Supplementary figure 1: Staining patterns of the 117 triple-negative breast cancers investigated (DOCX 22 kb)
