# Supplementary material for: TRPS1 expression in cytokeratin 5 expressing triple negative breast cancers, its value as a marker of breast origin
Source: Virchows Arch. 2023 Apr 3;482(5):861–8. doi: 10.1007/s00428-023-03535-4 (PMC10156897; doi:10.1007/s00428-023-03535-4)
Supplement: Supplementary file 1 — Supplementary figure 1: Staining patterns of the 117 triple-negative breast cancers investigated (DOCX 22 kb) [file 428_2023_3535_MOESM1_ESM.docx]

Supplementary figure 1: Staining patterns of the 117 triple-negative breast cancers investigated

(Starting from 12 o’clock position, clockwise from TRPS1 only to none)
